# Thermal Anemometry Grid Sensor

**DOI:** 10.3390/s17071663

**Published:** 2017-07-19

**Authors:** Martin Arlit, Eckhard Schleicher, Uwe Hampel

**Affiliations:** 1 Technische Universitaet Dresden, AREVA Endowed Chair of Imaging Techniques in Energy and Process Engineering, 01062 Dresden, Germany; uwe.hampel@tu-dresden.de; 2 Helmholtz-Zentrum Dresden-Rossendorf, Institute of Fluid Dynamics, P.O. Box 510119, 01314 Dresden, Germany; e.schleicher@hzdr.de

**Keywords:** temperature measurement, thermal anemometry, grid sensor

## Abstract

A novel thermal anemometry grid sensor was developed for the simultaneous measurement of cross-sectional temperature and axial velocity distribution in a fluid flow. The sensor consists of a set of platinum resistors arranged in a regular grid. Each platinum resistor allows the simultaneous measurement of fluid temperature via electrical resistance and flow velocity via constant voltage thermal anemometry. Cross-sectional measurement was enabled by applying a special multiplexing-excitation scheme. In this paper, we present the design and characterization of a prototypical sensor for measurements in a range of very low velocities.

## 1. Introduction

The measurement of local fluid velocities is a very common task in scientific and engineering research, where a number of different principles and techniques are available. Among them are local probes, non-invasive point measuring techniques (e.g., laser Doppler anemometry), and non-invasive field measuring techniques (e.g., particle imaging velocimetry, PIV). Local probes for point velocity measurements such as hot wire anemometer probes, pitot tubes, or vane anemometers are mostly used when the measuring effort needs to be low or optical access is not properly given. A workaround associated with higher effort is the spatially resolved measurement of the velocity field. For scientific and engineering flow measurement, optical measurement techniques such as PIV have been established [1]. However, they are sometimes not applicable (e.g., when the fluid is opaque or when there is restricted or no optical access to the flow field). In such cases, one possible alternative is the traversing of single sensors [2]; however, aside from the higher effort required for the mechanical traversing, the asynchronous sampling of the flow field might also be inappropriate in some cases. However, synchronous spatial sampling by a multitude of sensors or sensor rakes is often unacceptable in terms of complexity and intrusiveness.

A novel grid sensor for the spatially distributed measurement of axial flow velocity via thermal anemometry is presented in this paper [3]. The development originated from the issue of measuring gas phase axial velocity in a number of flow channels within a heated tube bundle in a thermal hydraulic test facility with no optical and restricted mechanical access. The utilization of a special multiplexing electronic circuit instead of multichannel electronics allowed for the synchronous operation of N × M single thermal anemometry sensors in a grid-like arrangement (with only N transmitters and M receivers) that supplies excitation voltages and leads the measurement signal (an electrical current), respectively. A prototypical sensor with nine measuring points was built and tested. This paper describes the sensor design, including the selection of suitable thermal anemometry operation modes, as well as a sensor characterization with an exemplary measurement of the gas flow profile in a pipe.

## 2. Grid Sensor Technique

**Figure 1:**
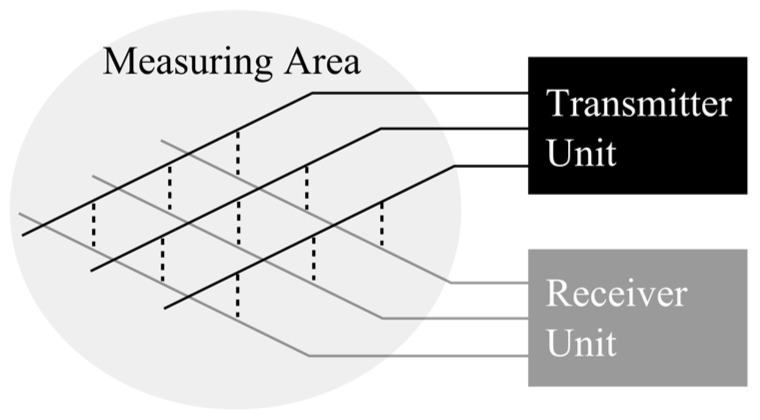
Functional principle of multiplexed grid-type sensors.

There are two ways to arrange a multitude of sensors for a distributed or field measurement of a physical parameter. One way is to directly connect each sensor to processing electronics, and the other is to use a multiplexing scheme. The latter is clearly preferred since the wiring effort and flow field obstruction are lower. However, the latter also requires that the sensor element is sensitive in an electrical property that can be measured with a voltage or current excitation scheme. This has some consequences, as it is not possible to apply the well-known principle of constant-temperature anemometry. As shown in Figure 1 it is very common and straightforward to arrange sensors in a planar regular grid; however, other topologies are possible as well.

In electrical grid sensors, each cross-point sensor element is typically excited by a voltage on one of the connecting wires (transmitter) while the other connecting wire (receiver) is used to measure the transmitted electrical current; furthermore, current excitation and voltage measurement is also possible. Moreover, DC and AC excitation schemes can be used, of which the most prominent is the wire-mesh sensor where wires form a simple grid with no further sensor elements, and the electrical properties of the fluid in the wire crossings can be measured in this way. Measuring electrical conductivity gives rise to a conductivity wire-mesh sensor [4], while an AC-based capacitance measuring scheme can be used for non-conducting fluids [5]. Hence, for a wire-mesh sensor, the fluid volume in the wire crossing is itself the sensor element. While wire-mesh sensors can measure phase fractions (e.g., in gas/water or gas/oil flows), they cannot directly measure continuous phase velocities. 

Grid arrangements with passive resistive, inductive, or capacitive elements in the crossing points are known from, for example, computer keyboards, touch screen displays, magnetic core memories, and other applications. Schaefer et al. [6] and Ritterath et al. [7] developed sensors with resistive sensor elements to measure the temperature distributions on surfaces and in flow cross-sections.

## 3. A Brief Review of the Principles of Thermal Anemometry

A resistor with temperature-dependent resistance exposed to a fluid can be used to both measure fluid temperature and flow velocity. The latter is possible by evaluating the heat flux from the resistor to the fluid. This principle is known as thermal anemometry. The temperature can be recalculated from measured resistance if an unambiguous relationship between both exists. For metal resistors with a monotonic functional dependency, this is given, for example, via a polynomial equation of the form

(1) $$R\left(\vartheta_{s}\right)=R_{0}\times \left[1+A_{\vartheta }\times \left(\vartheta_{s}-\vartheta_{0}\right)+B_{\vartheta }\times {\left(\vartheta_{s}-\vartheta_{0}\right)}^{2}\right]$$

with the nominal resistance $R_{0}$ at the reference temperature $\vartheta_{0}$ and temperature coefficients, e.g., for platinum, $A_{\vartheta }=3.9083\;\times \;10^{-3\;}{}^{\circ}C^{-1}$ and $B_{\vartheta }=-5.775\;\times \;10^{-7}\;{}^{\circ}C^{-2}$. Measurement of resistance usually requires applying a voltage $U_{T}$ to the resistor and measuring an electrical current I via Ohm’s law

(2) $$R=\frac{U_{T}}{I}.$$

For temperature measurement and the electrical current, respectively, the voltage $U_{T}$ must be sufficiently small to avoid excessive self-heating given the Joule heating power

(3) $$P_{\mathrm{El}}=RI^{2}=\frac{{U_{T}}^{2}}{R}.$$

**Figure 2:**
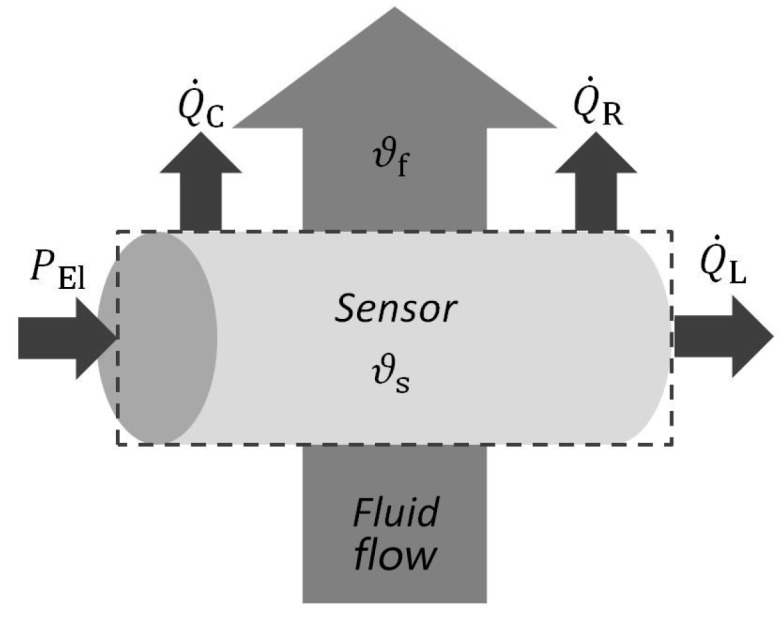
Heat transfer mechanisms in thermal anemometry principle.

However, this effect is being used for thermal anemometry in Reference [8]. The resistor was then heated by applying a sufficiently high voltage $U_{H}$. The heat is released into the fluid, which gives the power balance

(4) $$m\times c_{p}\times \dot{\vartheta }=P_{\mathrm{El}}-{\dot{Q}}_{C}-{\dot{Q}}_{L}-{\dot{Q}}_{R}$$

with the mass m and the specific thermal capacity $c_{p}$ of the resistor, the temporal gradients of the sensor temperature $\dot{\vartheta }$, the convective heat flux ${\dot{Q}}_{C}$, the conductive heat flux ${\dot{Q}}_{L}$, and the radiative heat flux ${\dot{Q}}_{R}$ (Figure 2). The dominating mechanism is the convective heat transfer

(5) $${\dot{Q}}_{C}=\alpha \times A\times \Delta \vartheta$$

with the heat transfer coefficient α, the sensor surface A and the overheating $\Delta \vartheta =\vartheta_{s}-\vartheta_{f}$ (i.e., the temperature difference between the sensor surface,$\;\vartheta_{s}$, and the fluid,$\;\vartheta_{f})$. The convective heat transfer occurs via both thermally-induced free convection and forced convection. The overheating should be adjusted in such a way that the latter is dominating. Therefore, the correlation $Re\;>\;Gr^{1/3\;}$ of Collis and Williams [9] with the Reynolds number Re=Re(v) and the Grashof number Gr=Gr(Δϑ) has to be observed. This means that overheating should be kept as low as possible to reduce the influence of buoyancy effects on the measurement at small fluid velocities. Forced convection is typically expressed via the dimensionless Nusselt number Nu; that is,

(6) $$Nu=\alpha \times \frac{l}{\lambda },$$

where λ denotes the thermal conductivity of the fluid and l a characteristic length. There are numerous correlations for the Nusselt number for fluid flow around cylinders of infinite length; for example, from Collis and Williams [9] or Kramers [10].

There are four operation modes for thermal anemometry, which are summarized in Table 1. In the constant temperature mode, the sensor resistance R and temperature $\vartheta_{s}$, respectively, are controlled by adjusting the applied voltage $U_{H}$. The calibration curve is in the simplest form $U_{H}=V\;+\;Wv^{0.5}$ with the flow velocity v and the empirical calibration parameters V and W [11]. Non-controlled operation modes are at constant current and constant voltage anemometry [12]. There, the applied current and voltage are kept constant, respectively. The sensor will then obtain an equilibrium temperature $\vartheta_{s}$. From the overheating Δϑ, the flow velocity can be correlated with a calibration curve. An unsteady method is thermal transient anemometry [13]. One operation cycle consists of two phases: first, the sensor is heated by applying a heating voltage; and second, the sensor temperature is measured by driving a sensing current through the resistor. During the second phase, the sensor cools down and the fluid velocity is determined from the time constant τ of the temperature drop measured via the sensor voltage for constant current. Independent of the operation mode, the flow velocity is usually determined via calibration data.

## 4. Thermal Anemometry Grid Sensor (TAGS)

### 4.1. General Sensor Design and Applicable Operation Modes

**Figure 3:**
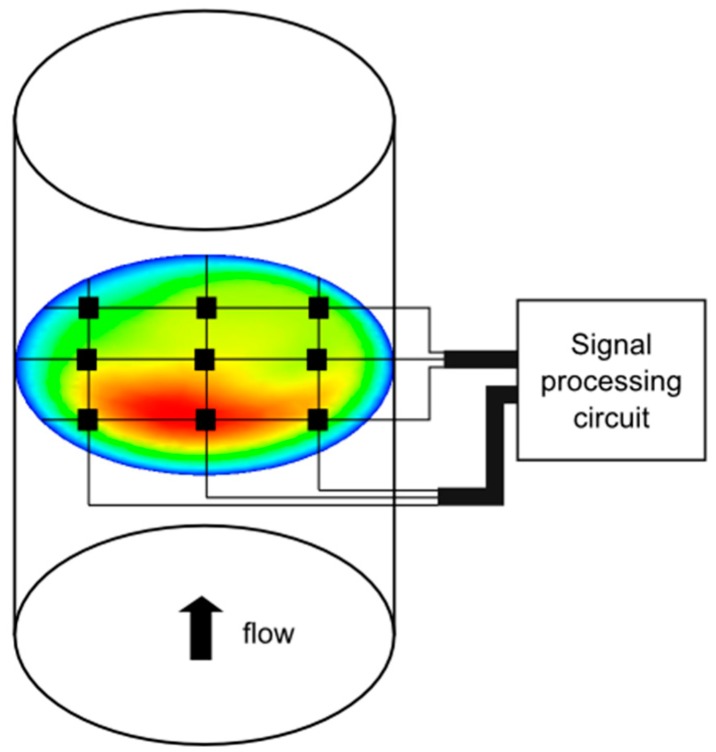
Scheme of a thermal anemometry grid sensor in a pipe cross-section (velocity color scale: blue/low to red/high).

The fundamental concept of the thermal anemometry grid sensor is the positioning of single resistors with a temperature-dependent resistance in a grid-like arrangement. This is schematically shown in Figure 3 for a 3 × 3 configuration. A grid-like support structure or frame holds the resistors in place and carries the electrical wires required for the multiplexing scheme. 

The operation modes for thermal anemometry presented in Section 3 are for single probes, but are not as straightforwardly applicable for a resistor network, as the one proposed here. A kind of addressing scheme is required for the operation of the resistors in the grid, and is discussed below. 

**Figure 4:**
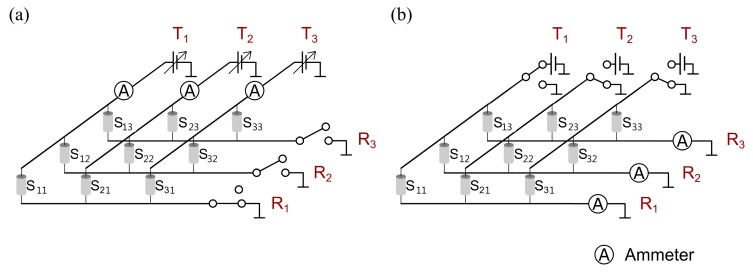
Possible TAGS addressing schemes: (**a**) Scheme 1; and (**b**) Scheme 2.

In principle, all operation modes are applicable. To gain access to each resistor individually under the boundary condition of measuring voltages and electrical currents at the transmitter/receiver wires, two addressing schemes are eligible and are summarized in Table 2. In Scheme 1, the resistors are addressed line-by-line by setting the addressed receiver line to ground potential while the other receivers are set to a high-impedance state (Figure 4a). The voltages at the transmitters can be adjusted individually. The currents have to be measured on the transmitter side to assign it to a specific resistor. In contrast, the resistors are addressed column-by-column by applying a constant voltage to the transmitters of the activated column while the other transmitters and the receivers are set to ground (Figure 4b) in Scheme 2. The currents are measured on the receiver side (e.g., via transimpedance amplifiers). One disadvantage of this scheme (for the constant temperature anemometry, CTA, and constant current anemometry, CCA, modes) is that the voltage for each resistor has to be controlled individually, which allows the interrogation of only one resistor element at a time. In both schemes, the addressing is realized via analog switches.

### 4.2. Analysis of the Applicable Operation Modes

The above-mentioned schemes were analyzed to prove the correct measurement of electrical quantities. The requirement for a suitable scheme is that the measured quantities $I_{m}$ or $R_{m}$ correspond to the true quantities at the sensors $I_{r}$ or $R_{r}$, respectively. The evaluation of the applicability of the schemes was calculated using LTSpice IV, a software program used to simulate electronic circuits.

Therefore, the circuit diagrams of both addressing schemes with Pt100 resistors ($R_{0}=100\;\Omega$ at $\vartheta_{0}=0\;{}^{\circ}C$) were implemented in the software. The unaddressed sensors were assumed to have a temperature of $\vartheta_{f}=16\;{}^{\circ}C$ that corresponded to a resistance of R=106 Ω. The voltages applied to the transmitters $U_{H}$ are presented in Table 3. In the simulation for Scheme 1, the true resistances $R_{r}$ were set to 120 Ω. The measured resistances $R_{m}$ were calculated from the voltage $U_{H}$ and the currents passing the ammeters. In Scheme 2, the resistances $R_{r}$ of the addressed column (transmitter T1) were randomly set and represented different equilibrium temperatures of the sensors.

For the given example in Scheme 1, the maximum relative error between the measured and the actual resistances was $\left(R_{m}-R_{r}\right)/R_{m}=10.8\%$ (Table 3). When the corresponding electrical currents were considered, a difference of ΔI=1.89 mA was noticed. In general, the reason that disables Scheme 1 are the parasitic currents over the unaddressed sensors due to differences in the voltages on the transmitters. In Scheme 2, the measured and the actual quantities were in good agreement, and the results have enabled the use of Scheme 2 in the TAGS thus far. Each single resistor has to be scanned individually in CTA and CCA operation mode, which makes them unsuitable for use in the TAGS. 

### 4.3. Electronic Circuit

**Figure 5:**
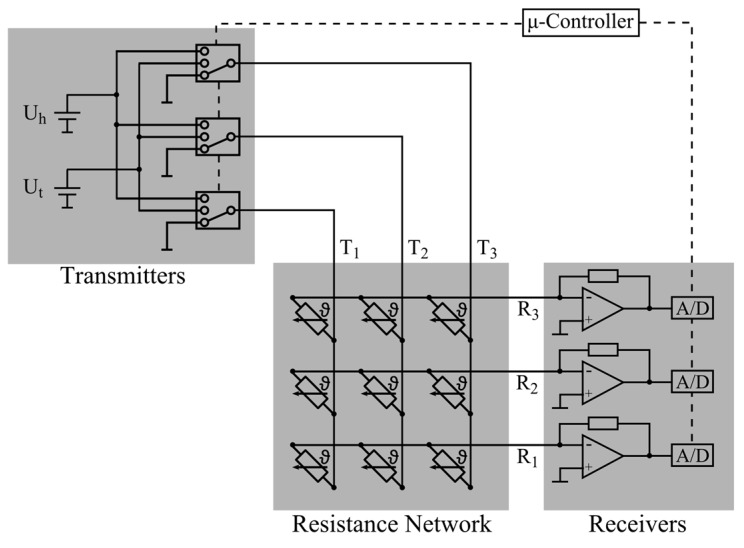
Principle sketch of the thermal anemometry grid sensor electronic circuit.

The electronics consist of transmitter units, receiver units, and the resistor network itself (Figure 5). The transmitter unit switched two voltage levels $U_{T}$ and $U_{H}$, as well as the ground potential to the transmitter wires of the resistor network by means of analog multiplexers. The measured signal was the electrical current at the receivers R*_j_*. At the receiver, currents were converted into corresponding voltages by transimpedance amplifiers. For data processing, these voltages were sampled by analog-to-digital converters. Both the control of the multiplexers and the processing of the digital data were performed with a microcontroller.

### 4.4. Operation Cycle

**Figure 6:**
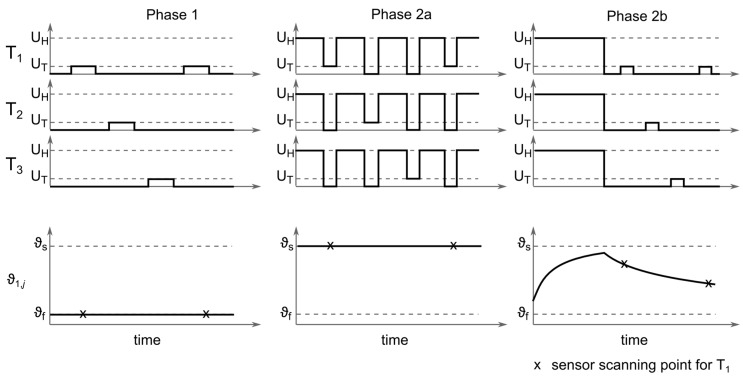
Timing diagram of the TAGS operation cycle, showing the voltages applied to the transmitters and temperature course of one resistor addressed by T_1_.

To determine the fluid velocity, the fluid temperature $\vartheta_{f}$ and the temperature of the heated sensor $\vartheta_{s}$ have to be measured. Therefore, two operation cycles were conducted consecutively:

1. First, $U_{T}$ was applied sequentially to the transmitters, and the fluid temperatures $\vartheta_{f}$ were determined via a temperature calibration curve from the measured currents.
2a. Next, for the constant voltage anemometry (CVA) mode, the overheat temperatures $\vartheta_{s}$ of the resistors in the equilibrium were of interest. Therefore, the voltage $U_{H}$ was simultaneously applied to all transmitters to heat the resistors. Subsequently, $U_{T}$ was applied sequentially to the transmitters to measure the temperatures $\vartheta_{s}$ in the overheated state (Figure **6**). It was ensured that cooling of the resistors during this unheated phase with $U_{T}$ was not too extensive. For the sensor reported in this paper, we targeted a temperature decrease smaller than 0.01⋅Δϑ.
2b. For the alternative thermal transient anemometry (TTA) mode, the resistors were heated simultaneously by application of $U_{H}$ to all transmitters. After a sufficient heating time, but not necessarily up to equilibrium temperature, the heating voltage was switched off and the time constant of the temperature drop during the cooling period was measured by the sequential application of $U_{T}$ to the transmitters. The ratio of the durations between the heated and the unheated phase as well as the resulting sampling time is dependent on the thermal relaxation time of the resistors.

## 5. Sensor Test and Characterization

### 5.1. Experimental Setup

**Figure 7:**
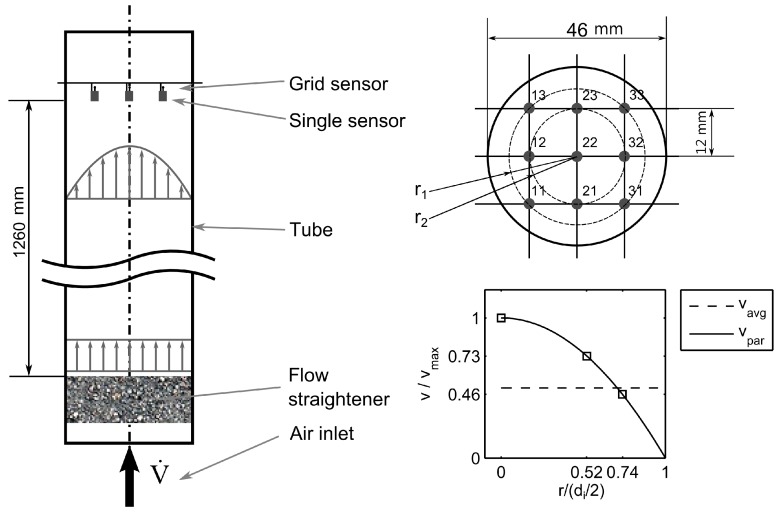
Experimental setup for a 3 × 3 TAGS in a pipe with laminar gas flow.

The functionality of the thermal anemometry grid sensor was tested in a laminar tube flow scenario. For such, the velocity profile is theoretically known as

(7) $$v_{\mathrm{par}}=v_{\max}\left[1-{\left(\frac{r}{d_{i}/2}\right)}^{2}\right]$$

with $v_{\max}=\;2\cdot v_{\mathrm{avg}}$, the radial position r, and the tube diameter $d_{i}$ [14]. Experimental investigations were performed in a tube flow channel with an inner diameter of $d_{i}=46\;\mathrm{mm}$. An air flow at room temperature ($\vartheta_{f}=22\;{}^{\circ}C$) was controlled via a mass flow controller and varied between $\dot{V}=0\;\mathrm{SLPM}\;$and $\dot{V}=14\;\mathrm{SLPM}$ (reference condition ϑ = 25 °C, p = 101.3 kPa). This corresponded to a maximum average flow velocity of $v_{\mathrm{avg}}\;=\;0.14\;m/s$ and a Reynolds number of Re = 419 regarding the inner diameter $d_{i}$. The tube was orientated in a vertical direction. For flow homogenization, a flow straightener sponge was mounted at the inlet (Figure 7, left). To ensure a fully developed parabolic flow profile at the measurement plane, the hydrodynamic entrance length L = 1260 mm exceeded the recommended minimum entrance length of 1156 mm.

(8) $$L_{\min}=0.06\times Re\times d_{i}.$$

**Figure 8:**
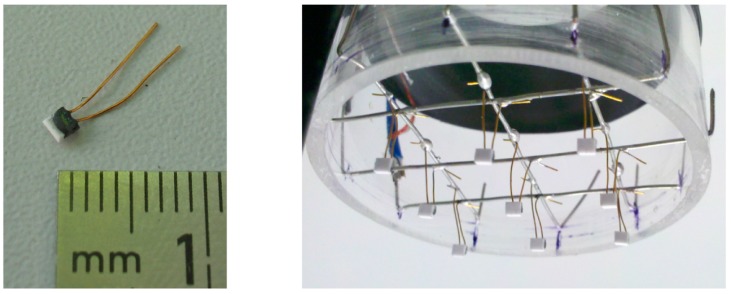
Photographs of the platinum film resistors (**left**) and 3 × 3 lab prototype of the TAGS (**right**).

The demonstrator TAGS was a 3-by-3 arrangement with nine sensor elements (Figure 7, top right and Figure 8). The center crossing point (22) was concentric to the tube. The distance between the parallel wires was l = 12 mm. The radial positions of the eccentric measurement points were $r_{1}/\left(d_{i}/2\right)\;=\;0.52$ and $r_{2}/\left(d_{i}/2\right)\;=\;0.74$. The platinum resistors had dimensions of 2 mm by 2 mm by 0.98 mm with a nominal resistance of $R_{0\;}=\;100\;\Omega$ at the reference temperature $\vartheta_{0}=0\;{}^{\circ}C$ where planar surfaces were orientated longitudinal to the flow direction. The theoretical flow velocities from Equation (7) were $v_{22}=\;v_{\max}$, $v_{r1}\;=\;0.73\cdot v_{\max}$, and $v_{r2}=0.46\cdot v_{\max}$ (Figure 7, bottom right). Applied voltages for temperature measurement were $U_{T}\;=\;0.13\;V$ and for heating $U_{H}=2.80\;V$. To exclude the influence of the wire resistances on the temperature measurement, the demonstrator TAGS was calibrated in a climate chamber in a range between $\vartheta_{f}=25\;{}^{\circ}C$ and $\vartheta_{f}=80\;{}^{\circ}C$ in steps of 5 K.

**Figure 9:**
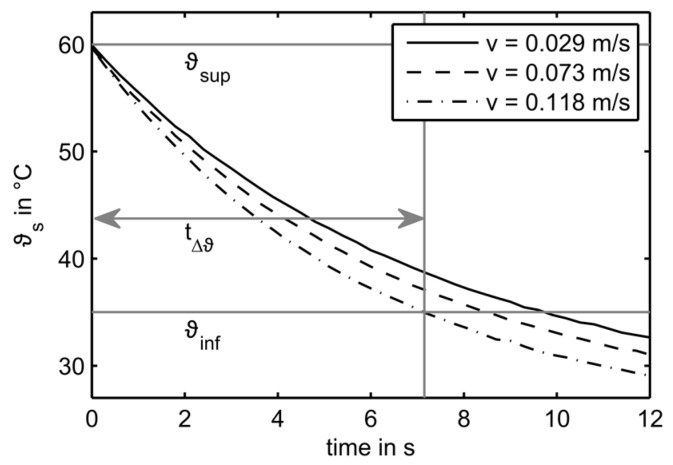
Example of temperature progress in the cooling phase for sensor S_33_ and three different flow velocities.

Using the TTA operation mode, the time constant τ can be determined by fitting an exponential function to the cooling curve; however, this is rather complex. Instead, the duration $t_{\Delta \vartheta }$ of the cooling curve passing through a threshold range was determined (Figure 9), as this may be uniquely related to velocity.

### 5.2. Modelling of the Sensor´s Heat Balance

As described in Section 3, the major heat dissipation mechanisms in thermal anemometry are heat conduction and convective heat transfer, where the latter includes a flow-dependent heat transfer coefficient for mixed convection [15]

(9) $$\alpha =\sqrt[{3}]{\alpha_{\mathrm{free}}^{3}+\alpha_{\mathrm{forced}}^{3}}\;.$$

With respect to the application in low velocity ranges, both the free and forced convection must be considered

(10) $$\alpha_{\mathrm{free}}=Nu_{\mathrm{free}}\frac{\lambda }{l_{\mathrm{free}}}$$

(11) $$\alpha_{\mathrm{forced}}=Nu_{\mathrm{forced}}\frac{\lambda }{l_{\mathrm{forced}}}$$

with the thermal conductivity λ of the fluid, the characteristic lengths $l_{\mathrm{free}}$, and $l_{\mathrm{forced}}$ and the corresponding Nusselt number $Nu_{\mathrm{free}}$ and $Nu_{\mathrm{forced}}$. The crucial point in calculating the convection is the application of the correct empirical Nusselt number correlation. Since the planar surfaces of the platinum resistors were oriented vertically, one may use the Nusselt correlation for a vertical wall [16]:

(12) $$Nu_{\mathrm{free}}={\left[0.825+0.387{\left(Ra\times f_{1}\right)}^{\frac{1}{6}}\right]}^{2}$$

(13) $$f_{1}={\left[1+{\left(\frac{0.492}{\mathrm{Pr}}\right)}^{\frac{9}{16}}\right]}^{-\frac{16}{9}}$$

(14) $$Ra=Gr\times Pr=\frac{\rho^{2}g\beta \Delta \vartheta l_{\mathrm{free}}^{3}}{\mu^{2}}\times Pr$$

with the factor $f_{1}$ with the Prandtl number $Pr=\mu c_{p}/\lambda$; the Rayleigh number Ra that includes the density ρ; the gravitational constant g; the expansion coefficient $\beta =1/\left(273.15\;{}^{\circ}C+\vartheta_{f}\right)$ for gases, the dynamic viscosity μ; and the specific heat capacity $c_{p}$. For the calculation of the forced convection, the approach for a flow lengthwise to a planar wall was not applicable, since the model was not adaptable to the experimental data. Instead, the correlation

(15) $$Nu_{\mathrm{forced}}=2\times \frac{0.55}{K^{0.5}}+\frac{10}{9}\times \frac{0.95}{K^{0.1}}$$

for a flow lengthwise to cylinder was used [17], with the curvature parameter

(16) $$K=\frac{\mu }{\rho }\times \frac{l_{\mathrm{forced}}}{{\left(d/2\right)}^{2}}\times \frac{1}{v}\;.$$

Parameters are the flow velocity v and d, which is the diameter of the circular surface area of the cylinder adequate to the platinum resistor surface area normal to the flow direction.

Heat conduction from the heated sensor along the sensor supports was an undesirable heat dissipation mechanism. The platinum resistors were supported by two thin wires. The heat conduction through these wires is described by

(17) $${\dot{Q}}_{L}=\lambda_{w}\left(\frac{1}{x_{1}}+\frac{1}{x_{2}}\right)A_{w}\left(\vartheta_{s}-\vartheta_{f}\right)\;,$$

with the thermal conductivity $\lambda_{w}$ of the wire material; and the measured lengths $x_{1}$ and $x_{2}$ from the platinum resistor to the grid wires with the cross-sectional area $A_{w}$. It was assumed that the temperature of the grid wires was identical to the fluid temperature $\vartheta_{f}$. This assumption may be incorrect in the case of thermal gradients in the measurement plane. The influence on the measurement signal was expected to be negligible.

Putting Equations (3)–(5) and (17) together gives a balanced state ($\dot{\vartheta }=0$); e.g., in constant voltage anemometry (CVA) mode,

(18) $$0=\frac{U_{H}^{2}}{R}-\left[\alpha \left(v\right)A+\lambda_{w}A_{w}\left(\frac{1}{x_{1}}+\frac{1}{x_{2}}\right)\right]\cdot \Delta \vartheta .$$

With this model and the properties of the platinum resistors, as well as the operation conditions, a calibration curve Δϑ = Δϑ(v) can be calculated.

### 5.3. Comparison of Experimental Results and Modelling

**Figure 10:**
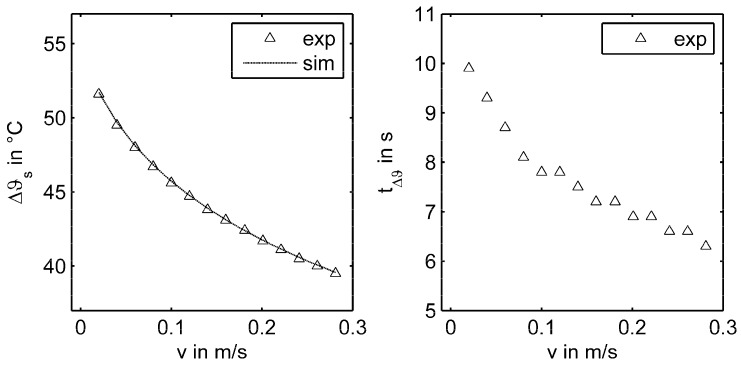
Overheating Δϑ of the sensor in the CVA mode (**left**) and cooling time in TTA mode (**right**) for the investigated velocity range of the center sensor element S_22_.

The results for both CVA and TTA operation modes of the central sensor element S_22_ are shown in Figure 10. On the left side for CVA, Δϑ was plotted as a function of the flow velocity. With increasing flow velocity v, the overheating Δϑ decreased. Aside from the experimental results, the simulated characteristic curve resulting from the presented heat balance model for sensor S_22_ was plotted. It was evident that the curve calculated from Equation (18) was in very good agreement with the experimental data. On the right side of Figure 10, the characteristic cooling times $t_{\Delta \vartheta }$ (determined via the TTA mode) for the same velocity points were plotted. With increasing flow velocity v, the cooling time $t_{\Delta \vartheta }$ decreased.

**Figure 11:**
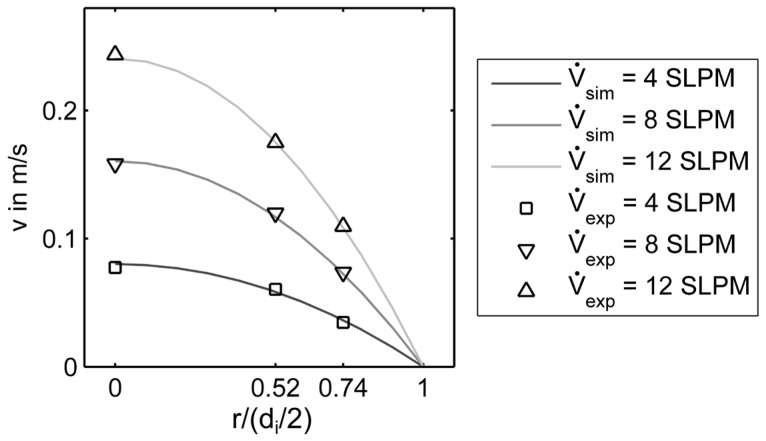
Measured velocities compared to the expected parabolic flow profile for three different volumetric flow rates.

With the model, a set of calibration curves for all sensors S*_ij_* at the radial positions $r_{1}$ and $r_{2}$ were calculated by adjusting $x_{1}$ and $x_{2}$. Thereby, the flow velocity was calculated from the measured overheating Δϑ. In Figure 11, the theoretical parabolic velocity profiles calculated from Equation (7) were plotted for three different volumetric flow rates. Furthermore, the determined velocities from the measured Δϑ of the sensors at three radial positions are shown. This demonstrated that the flow profile in the pipe was measured correctly with the prototypical TAGS.

Each measured parameter in Equation (18) had an uncertainty. The individual influence on the determined velocity is presented in Table 4, and resulted in an overall uncertainty of 6.7%. The performance capability strongly depends on the applied voltage $U_{H}$. For shifting the sensitivity of a given calibration curve in a higher range of velocities, $U_{H}$ has to be increased.

Considering the response time of $t_{63\%}=4.9\;s$ (air flow, v=1 m/s) of the used platinum resistors, the demonstrator TAGS was suitable for stationary or slowly varying flow fields. Applying smaller platinum resistors with lower response times may increase the temporal resolution.

One potential application is the characterization of novel heat exchanger geometries by measuring the uniformity of the inflow condition upstream and the distributed temperature and flow field downstream of the heat exchanger. A more industrial application may be in air conditioning by the determination of an area averaged velocity from a spatially distributed point measurement. 

## 6. Conclusions

In this paper, a novel instrument for a spatially-distributed measurement of axial flow velocities was presented. Several single platinum resistors were positioned in a grid-like arrangement and were contacted in a wire grid. A special excitation-sampling scheme allowed the synchronous measurement of the flow velocities at all measurement points by the application of the thermal anemometry method. Realized operation modes were constant voltage anemometry (CVA) and thermal transient anemometry (TTA). Applicability was demonstrated by measuring the parabolic velocity profile in a laminar pipe flow for a range of very low velocities with an uncertainty of 6.7%. The general fields of application are flows with a pronounced 2D flow profile where other field measurement techniques (e.g., PIV) are less applicable.

## Figures and Tables

**Table 1:** Overview of known thermal anemometry operation modes.

Anemometry Operation Mode	Abbreviation	Characteristic Variable
Constant Temperature	CTA	$U=U_{H}\left(v\right)$
Constant Current	CCA	$U=U_{H}\left(v\right)$
Constant Voltage	CVA	$I=I_{H}\left(v\right)$
Thermal Transient	TTA	$\tau =\tau_{\mathrm{cool}}\left(v\right)$

**Table 2:** Addressing schemes for thermal anemometry grid sensor (TAGS) data acquisition (with reference to Figure 4).

Scheme	Number of Active Transmitters	Side of Current Measurement	Number of Simultaneous Scanning Points				
			$\vartheta_{f}$	CTA	CCA	CVA	TTA
1	3	Transmitter	3	3	3	3	3
2	1	Receiver	3	1	1	3	3

**Table 3:** Results of the evaluation of both addressing schemes described in the text.

Scheme	Nr.	$U_{H}$	$I_{m}$	$R_{m}$	$I_{r}$	$R_{r}$	ΔR/R
		V	mA	Ω	mA	Ω	
1	S_11_	2.1	15.61	134.5	17.50	120.0	10.8%
	S_21_	2.2	18.33	120.0	18.33	120.0	0.0%
	S_31_	2.3	21.05	109.3	19.16	120.0	−9.9%
2	S_11_	2.2	17.78	123.7	17.82	123.5	0.2%
	S_12_	0	18.25	120.5	18.82	120.4	0.2%
	S_13_	0	18.70	117.6	18.73	117.5	0.2%

**Table 4:** Uncertainty of measured parameters and the influence on the velocity.

Parameter	Value	Uncertainty	dv/v
$U_{H}$	2.80 V	0.01 V	4.0%
$x_{1}$	6.0 mm	0.1 mm	2.5%
$x_{2}$	7.9 mm	0.1 mm	1.5%
Δϑ	40.0 K	0.3 K	−4.6%
